# Depressive and Anxiety Symptoms and Their Comorbidity Across Industries: Multi-Industry Cross-Sectional Study in China

**DOI:** 10.2196/91782

**Published:** 2026-08-21

**Authors:** Lei Cao, Ruihong Ran, Huadong Zhang, Heling Bao

**Affiliations:** 1 Institute of Public Health and Safety Surveillance, Chongqing Center for Disease Control and Prevention, Chongqing, China; 2 Institute of Medical Information, Chinese Academy of Medical Sciences and Peking Union Medical College, No.3, Yabao Road, Chaoyang District, Beijing, China, 86 01052328688

**Keywords:** depression, anxiety, comorbidity, occupational stress, epidemiology

## Abstract

**Background:**

Depressive and anxiety symptoms are primary drivers of China’s mental health burden, with workplace psychological issues growing increasingly pronounced. However, evidence on industry-specific patterns of depressive and anxiety symptoms and their occupational determinants remains limited. Understanding these sector-specific differences is critical for pinpointing high-risk occupations and designing effective, tailored intervention strategies.

**Objective:**

Our objective was to assess the prevalence, determinants, and industry-specific patterns of depressive, anxiety, and comorbid symptoms across multiple industries.

**Methods:**

We conducted a cross-sectional survey among 10,112 workers from 11 industries in all districts or counties of Chongqing, China. Depressive and anxiety symptoms were measured using the 9-item Patient Health Questionnaire (PHQ-9) and the 7-item Generalized Anxiety Disorder scale (GAD-7), with comorbidity defined as meeting criteria for both. Weighted prevalence and 95% CIs were calculated using Taylor series linearization accounting for cluster effects. Mixed-effects logistic regression models were applied to examine associations between demographic, health, and occupational factors and depressive and anxiety symptoms, as well as to compare these outcomes across industries. Multinomial mixed-effects models were used to examine patterns of comorbidity by modeling the 4-category outcome as follows: no symptoms, depression only, anxiety only, and comorbid depressive-anxiety symptoms.

**Results:**

Overall, 19.0% (95% CI 16.7%‐21.3%) of workers reported depressive symptoms, 7.8% (95% CI 6.4%‐9.1%) reported anxiety symptoms, and 5.9% (95% CI 4.8%‐7.1%) experienced depressive-anxiety comorbidity. Younger age, being single, recent sick leave, multisite bodily pain, working hours, occupational stress, and health literacy were strongly associated with all outcomes, with the largest effects observed for the comorbid symptoms. Significant disparities were observed across industries, with the highest prevalence in education, health care, express delivery, and construction, and the lowest in traditional manufacturing and mining. These disparities persisted significantly after comprehensive adjustment for demographic, health-related, and occupational factors.

**Conclusions:**

Depressive, anxiety, and comorbid symptoms are prevalent among Chinese workers, exhibiting significant variation across industries and distinct links to modifiable occupational and health factors. Targeted, sector-specific interventions and integration of mental health promotion into occupational health systems are vital to alleviate work-related psychological burdens and propel national mental health initiatives.

## Introduction

Depression and anxiety disorders have emerged as leading contributors to the global burden of disease, with the prevalence substantially increasing since 2010 and exacting a heavy toll on individuals and society through suffering, reduced productivity, and economic costs [1]. Recent global estimates suggest a continuing rise in disability-adjusted life years due to these disorders [2]. In China, meta-analytic data indicate that up to 31% of the population experience depressive symptoms and 29% report anxiety symptoms [3]. Critically, comorbidity between depression and anxiety is common [4]. Individuals suffering from both tend to have greater functional impairment and poorer quality of life than those with only one condition [5,6]. This convergence of high prevalence and co-occurrence has intensified the public health urgency.

A substantial body of evidence demonstrates that work-related factors—including long working hours, high job demands, low job control, inadequate social support, and emotional labor—are consistently associated with elevated risks of depression and anxiety across diverse occupational settings [7-9]. As these psychosocial risks have become increasingly recognized, many countries have incorporated work-related mental health into policy frameworks. For example, the European Union has mandated psychosocial risk management within occupational safety regulations to safeguard workers’ psychological well-being [10], while the United States has implemented initiatives targeting work stress, fatigue, and organizational climate [11].

China has also elevated mental health to a national priority. The Healthy China Action (2019‐2030) plan includes goals to reduce the prevalence of depression and anxiety and strengthen access to mental health services [12]. However, the mental health impact of occupational exposures has received far less scrutiny [7,13]. Evidence on industry-specific risks, patterns of depressive and anxiety symptoms, and work-related determinants remains limited. Industry-level data are urgently needed to inform targeted interventions and guide the development of occupational mental health prevention strategies.

This study aimed to assess depressive symptoms, anxiety symptoms, and their comorbidity across multiple industries using a large, representative sample of workers in Chongqing, China. Our objective was to identify industry-specific significant differences in the prevalence of mental health outcomes and to examine the demographic, health-related, and occupational factors associated with them. In addition, we sought to characterize the patterns of comorbidity between depression and anxiety across industries, providing evidence to inform targeted workplace mental health interventions.

## Methods

### Study Design

This cross-sectional study was a part of the 2024 Occupational Health Literacy Surveillance Program in Chongqing, China. We followed the STROBE (Strengthening the Reporting of Observational Studies in Epidemiology) reporting guideline and checklist to draft this manuscript (Checklist 1).

### Survey Development

From January 1 to December 31, 2024, we conducted a survey to assess occupational health literacy, behavior, and mental health among urban workers across all 38 districts/counties in the Chongqing province (Multimedia Appendix 1). According to the industrial characteristics and Industrial Classification for National Economic Activities (GB/T 4754‐2017), we selected 11 industries from the secondary and tertiary sectors, including the leather, fur, feather and their products, and footwear manufacturing industry (C-1900), automotive manufacturing industry (C-3600), electrical equipment manufacturing industry (C-3800), construction industry (E-0000), non-metallic mineral mining industry (B-1000), computer and communication equipment manufacturing industry (C-3900), health care and social work industry (Q-8400), education industry (P-8300), environmental sanitation industry (N-7820), transportation industry (G-5400), and express delivery and food delivery industry (G-6000).

### Participant Recruitment

Eligible participants were front-line workers aged 16‐59 years with at least 6 months of work experience in the selected industries, whereas administrative staff were excluded. We applied a multistage stratified cluster sampling design and at least 800 workers were required in each industry. For the secondary industries, enterprises served as primary sampling units and were stratified by size (large, medium, small/micro). Within each stratum, enterprises were randomly selected, and target sample sizes were allocated proportionally, with approximately 160, 240, and 400 workers recruited from large, medium, and small/micro enterprises, respectively. Large enterprises were limited to no more than 10% of the sampled worksites. For the tertiary industries, 5 districts (Yuzhong, Banan, Kaizhou, Jiulongpo, and Yongchuan) were selected, and industry-specific clusters were sampled within each district. Health care workers were recruited from 1‐2 tertiary/secondary hospitals and 2‐3 primary hospitals per district; teachers were sampled from 1‐3 middle schools and 1‐3 primary schools; sanitation workers from 1‐3 environmental service agencies; transportation workers from 2‐3 taxi, bus, freight, or ride-hailing companies; and delivery workers from 1‐3 courier or food-delivery companies. Each district aimed to recruit at least 200 participants per industry. Finally, 10,112 workers were invited to completed an anonymous, self-administered electronic questionnaire.

### Survey Measures

The primary outcomes were the prevalence of depressive symptoms, anxiety symptoms, and their comorbidity. Depression and anxiety were assessed using the validated Chinese versions of the 9-item Patient Health Questionnaire (PHQ-9) and the 7-item Generalized Anxiety Disorder scale (GAD-7) [14,15]. The PHQ-9 evaluates depressive symptoms over the past 6 months, and the GAD-7 assesses anxiety symptoms over the past 2 weeks. Both use a 4-point Likert scale and have demonstrated strong reliability and validity in Chinese populations [16,17]. Higher scores indicate greater symptom severity. Clinically relevant depressive and anxiety symptoms were defined using a cutoff value of ≥10 on each scale, and comorbidity was defined as meeting both thresholds. In this study, the scale demonstrated a Cronbach α of 0.89 for PHQ-9 and 0.91 for GAD-7.

Occupational stress was measured using the Core Occupational Stress Scale [18], developed for Chinese occupational settings and assessing social support, remuneration, dedication, and autonomy. A total score ≥50 indicated occupational stress (Cronbach *α*=0.94). Workload indicators included weekly working hours and night or rotating shift work. Occupational health literacy—assessing legal knowledge, protective knowledge, skills, and healthy workplace behaviors—was measured according to the national surveillance protocol. Additional covariates included age, sex, marital status, education, sick leave days, and multisite bodily pain.

### Statistical Analysis

Categorical variables were summarized as frequencies and percentages, and continuous variables as median with interquartile ranges. Differences between groups were assessed using chi-square or Mann-Whitney *U* tests. Standardized prevalence estimates were derived using sampling weights to represent the occupational population aged 16‐59 years in Chongqing, with poststratification adjustments for age and sex based on the Seventh National Census. Crude prevalence was reported for sensitivity analysis. Variances and 95% CIs were calculated using Taylor series linearization accounting for cluster effects.

Generalized linear regression models were used to examine associations between industry types, occupational stress, workload, occupational health literacy, health status, and mental health outcomes, adjusting for demographic characteristics. Random intercepts for district/county were included to account for the hierarchical sampling structure. For the comorbid outcome, the joint distribution of depression and anxiety was modeled as a 4-category variable (none, anxiety only, depression only, comorbidity). A Bayesian multinomial mixed-effects logistic regression was fitted using the *brms* package in R with a categorical logit link, with parameter variances and 95% credible intervals derived from Hamiltonian Monte Carlo posterior sampling. Results were presented as odds ratios (ORs) with 95% CIs. To further explore factors that may account for occupational differences in mental health, we fitted a series of sequentially adjusted models by progressively introducing demographic, health-related, and occupational variables. Changes in industry-specific ORs across models were examined to evaluate the extent to which these factors accounted for the observed heterogeneity across industries. All analyses were conducted in R version 4.4.1 (R Foundation for Statistical Computing). Two-sided *P* values <.05 were considered statistically significant.

### Ethical Considerations

This study was approved by the Ethics Committee of the Chongqing Academy of Preventive Medicine (approval no. KY-2025-026-1) before participant recruitment. Written informed consent was obtained from all participants prior to questionnaire completion. Participation was voluntary, and all data were collected anonymously, stored securely, and analyzed in deidentified form to protect participant confidentiality. The study was conducted in accordance with the ethical principles of the Declaration of Helsinki.

## Results

### Participant Characteristics

A summary of participant characteristics is presented in Table 1. A total of 10,112 workers from 11 industries were included, comprising 5366 (53.0%) men and 4746 (47.0%) women. Women were more often employed in education and health care, while men predominated in transportation, construction, and mining sectors. The mean age was 40.5 (SD 10.3) years. Most participants were married, and two-thirds had degrees from senior middle school or a higher institution. About half of the participants were determined to have low occupational health literacy. Approximately a quarter of participants worked more than 55 hours per week, and 45.4% (n=4595) reported night or rotating shifts. One-fourth experienced occupational stress. Mean scores were 6.7 (SD 4.6) for PHQ-9 and 3.2 (SD 4.1) for GAD-7.

**Table 1.** Sociodemographic and occupational characteristics of participants from in a cross-sectional survey in Chongqing, China (cross-sectional survey, January 1-December 31, 2024; N=10,112), stratified by sex.

	Overall	Men	Women
Overall, n (%)	10,112 (100)	5366 (53.0)	4746 (47.0)
Age (years)			
Mean (SD)	40.5 (10.3)	40.9 (10.7)	40.0 (9.8)
Age group, n (%)			
16‐39	4957 (49.0)	2558 (47.7)	2399 (50.6)
40‐59	5155 (51.0)	2808 (52.3)	2347 (49.5)
Relationship status, n (%)			
Single	1692 (16.7)	1067 (19.9)	625 (13.2)
Married	7887 (78.0)	4003 (74.6)	3884 (81.8)
Other	533 (5.3)	296 (5.5)	237 (5.0)
Education, n (%)			
Junior middle school and lower	3772 (37.3）	2014 (37.5)	1758 (37.0)
Senior middle school	3928 (38.8)	2503 (46.7)	1425 (30.0)
Undergraduate	2412 (23.9)	849 (15.8)	1563 (32.9)
Sick leave days in the past year, n (%)			
None	7903 (78.2)	4266 (79.5)	3637 (76.6)
Less than 3 days	1461 (14.5)	719 (13.4)	742 (15.6)
More than 3 days	748 (7.4)	381 (7.1)	367 (7.7)
Weekly working hours, n (%)			
35‐40 hours	2400 (23.7)	1193 (22.2)	1207 (25.4)
40‐55 hours	5258 (52.0)	2613 (48.7)	2645 (55.7)
Over 55 hours	2454 (24.3)	1560 (29.1)	894 (18.8)
Multisite bodily pain, n (%)			
None	4494 (44.4)	2621 (48.8)	1873 (39.5)
Less than 3	3355 (33.2)	1669 (31.1)	1686 (35.5)
More than 3	2263 (22.4)	1076 (20.1)	1187 (25.0)
Night shift, n (%)			
Yes	4595 (45.4)	2791 (52.0)	1804 (38.0)
No	5517 (54.6)	2575 (48.0)	2942 (62.0)
Occupational stress, n (%)			
Yes	2484 (24.6)	1449 (27.0)	1035 (21.8)
No	7628 (75.4)	3917 (73.0)	3711 (78.2)
Occupational health literacy, n (%)			
Low	5165 (51.1)	2716 (50.6)	2449 (51.6)
High	4947 (48.9)	2650 (49.4)	2297 (48.4)
Industry categories, n (%)			
P-8300: education industry	946 (9.4)	221 (4.1)	725 (15.3)
Q-8400: health care and social work industry	941 (9.3)	179 (3.3)	762 (16.1)
G-6000: express delivery and food delivery industry	917 (9.1)	668 (12.5)	249 (5.3)
G-5400: transportation industry	866 (8.6)	712 (13.3)	154 (3.2)
N-7820: environmental sanitation industry	888 (8.8)	257 (4.8)	631 (13.3)
C-3600: automotive manufacturing industry	919 (9.1)	631 (11.8)	288 (6.1)
C-3800: electrical equipment manufacturing industry	933 (9.2)	372 (6.9)	561 (11.8)
C-3900: computer and communication equipment manufacturing industry	921 (9.1)	417 (7.8)	504 (10.6)
B-1000: non-metallic mineral mining industry	890 (8.8)	757 (14.1)	133 (2.8)
C-1900: leather, fur, feather and their products, and footwear manufacturing industry	953 (9.4)	405 (7.6)	548 (11.6)
E-0000: construction industry	938 (9.3)	747 (13.9)	191 (4.0)
Mental health measures, mean (SD)			
PHQ-9^a^ score	6.7 (4.6)	6.8 (4.7)	6.6 (4.5)
GAD-7^b^ score	3.2 (4.1)	3.0 (4.1)	3.3 (4.1)

a PHQ-9: 9-item Patient Health Questionnaire.

b GAD-7: 7-item Generalized Anxiety Disorder scale.

### Prevalence of Depressive and Anxiety Symptoms

The standardized and crude prevalence of depressive symptoms, anxiety symptoms, and their comorbidity is detailed in Table 2 and Table S1 in Multimedia Appendix 2, respectively. Overall, 19.0% (95% CI 16.7%‐21.3%) of workers reported depressive symptoms, 7.8% (95% CI 6.4%‐9.1%) reported anxiety symptoms, and 5.9% (95% CI 4.8%‐7.1%) experienced comorbid depressive-anxiety symptoms. Younger workers (16‐39 years) and single individuals consistently exhibited higher comorbidity prevalence than their comparison groups. Participants who had more sick leave days had a markedly greater burden of comorbidity compared with those reporting less days or none (16.0% vs 10.1% and 4.2%, respectively). The prevalence of depressive and anxiety symptoms increased in a graded manner with longer working hours, more sites of body pain, night or rotating shifts, and higher scores of occupational stress. In addition, workers with lower occupational health literacy showed substantially elevated comorbidity prevalence compared with their counterparts (7.7% vs 4.1%).

**Table 2.** Weighted prevalence of depressive symptom, anxiety symptom, and comorbid symptoms among participants in Chongqing, China (cross-sectional survey, January 1-December 31, 2024; N=10,112), overall and by subgroups.

	Depressive symptoms, % (95% CI)	Anxiety symptoms, % (95% CI)	Comorbid symptoms, % (95% CI)		
			Depression only	Anxiety only	Comorbidity
Overall	19.0 (16.7‐21.3)	7.8 (6.4‐9.1)	13.1 (11.7‐14.5)	1.8 (1.5‐2.1)	5.9 (4.8‐7.1)
Sex					
Men	19.3 (16.7‐21.8)	7.7 (6.3‐9.1)	13.4 (11.7‐15.2)	1.9 (1.4‐2.3)	5.8 (4.7‐6.9)
Women	18.7 (16.1‐21.2)	7.9 (6.3‐9.4)	12.5 (11.2‐13.9)	1.7 (1.4‐2.0)	6.1 (4.6‐7.6)
Age group (years)					
16‐39	23.8 (21.6‐26.0)	9.9 (8.1‐11.6)	16.0 (14.6‐17.4)	2.0 (1.6‐2.4)	7.8 (6.3‐9.4)
40‐59	14.7 (12.3‐17.2)	5.9 (4.7‐7.1)	10.5 (8.9‐12.0)	1.6 (1.3‐2.0)	4.3 (3.2‐5.3)
Relationship status					
Single	26.5 (23.9‐29.1)	9.8 (8.3‐11.3)	18.3 (16.3‐20.4)	1.7 (1.2‐2.2)	8.1 (6.8‐9.5)
Married	16.7 (14.5‐18.9)	6.9 (5.6‐8.2)	11.6 (10.3‐12.9)	1.8 (1.5‐2.1)	5.1 (4.0‐6.2)
Other	23.8 (19.3‐28.2)	11.8 (7.5‐16.1)	14.1 (10.2‐17.9)	2.1 (0.8‐3.3)	9.7 (5.5‐13.9)
Education					
Junior middle school and lower	13.9 (11.9‐15.9)	6.3 (5.0‐7.5)	9.3 (8.2‐10.4)	1.7 (1.3‐2.0)	4.6 (3.6‐5.7)
Senior middle school	20.5 (17.9‐23.1)	7.8 (6.7‐8.9)	14.7 (12.6‐16.8)	2.0 (1.5‐2.5)	5.8 (4.9‐6.7)
Undergraduate	25.0 (23.3‐26.6)	10.1 (8.1‐12.2)	16.6 (15.5‐17.6)	1.7 (1.0‐2.5)	8.4 (6.9‐10.0)
Sick leave days in the past year					
None	15.4 (13.2‐17.7)	5.7 (4.5‐6.9)	11.2 (9.8‐12.6)	1.5 (1.2‐1.8)	4.2 (3.2‐5.3)
Less than 3 days	28.3 (25.4‐31.1)	12.8 (10.0‐15.5)	18.2 (15.7‐20.7)	2.7 (1.5‐3.8)	10.1 (7.9‐12.3)
More than 3 days	38.9 (35.7‐42.2)	19.7 (16.3‐23.1)	22.9 (20.0‐25.8)	3.7 (2.1‐5.2)	16.0 (13.0‐19.0)
Weekly working hours					
35‐40 hours	15.8 (13.3‐18.4)	6.6 (5.2‐8.0)	10.9 (8.9‐12.9)	1.7 (1.1‐2.3)	5.0 (3.8‐6.1)
40‐55 hours	17.8 (15.0‐20.6)	6.8 (5.3‐8.4)	12.5 (10.9‐14.2)	1.5 (1.2‐1.9)	5.3 (3.9‐6.6)
Over 55 hours	24.5 (21.2‐27.9)	10.8 (7.9‐13.7)	16.3 (13.9‐18.6)	2.5 (1.6‐3.4)	8.3 (6.0‐10.6)
Multisite bodily pain					
No	8.8 (7.5‐10.2)	3.0 (2.3‐3.8)	6.7 (5.6‐7.8)	1.0 (0.6‐1.3)	2.1 (1.5‐2.7)
Less than 3	21.9 (19.4‐24.5)	8.6 (7.3‐9.9)	15.5 (13.5‐17.5)	2.1 (1.5‐2.7)	6.5 (5.5‐7.4)
More than 3	35.5 (33.0‐37.9)	16.2 (14.3‐18.1)	22.4 (20.7‐24.2)	3.2 (2.6‐3.7)	13.0 (11.2‐14.9)
Night shift					
Yes	22.7 (19.9‐25.4)	9.5 (7.8‐11.2)	15.2 (13.3‐17.0)	2.1 (1.3‐2.8)	7.5 (6.2‐8.8)
No	17.2 (14.8‐19.5)	6.9 (5.6‐8.2)	12.0 (10.6‐13.4)	1.7 (1.5‐1.9)	5.2 (4.0‐6.3)
Occupational stress					
Yes	37.4 (34.2‐40.5)	17.6 (15.1‐20.0)	23.4 (21.1‐25.6)	3.5 (3.0‐4.1)	14.0 (11.6‐16.4)
No	13.0 (11.3‐14.6)	4.5 (3.7‐5.4)	9.7 (8.5‐10.9)	1.2 (0.9‐1.6)	3.3 (2.7‐3.9)
Occupational health literacy					
Low	21.9 (19.5‐24.2)	10.2 (8.9‐11.4)	14.2 (12.5‐15.9)	2.5 (2.1‐2.9)	7.7 (6.6‐8.7)
High	16.0 (13.3‐18.8)	5.2 (3.7‐6.8)	11.9 (10.1‐13.7)	1.1 (0.8‐1.4)	4.1 (2.9‐5.4)

Table 3 presents the associations of demographic, health, and occupational factors with depressive, anxiety, and comorbid symptoms in the fully adjusted mixed-effects models. Younger workers had consistently higher odds of depression only, anxiety only, and comorbid symptoms, and being single was associated with higher odds of depression only symptoms and comorbidity. Having more than 3 days of sick leave and experiencing pain at more than 3 body sites were strongly associated with all mental health outcomes, with the strongest associations observed for comorbid symptoms (OR 3.35, 95% CI 2.61‐4.29 and OR 5.66, 95% CI 4.36‐7.37, respectively). Occupational stress exhibited significant associations across all outcomes and was associated with approximately threefold higher odds of depression only (OR 2.99, 95% CI 2.62‐3.39) and anxiety only (OR 3.51, 95% CI 2.59‐4.76) symptoms, and nearly fivefold higher odds of comorbid symptoms (OR 4.78, 95% CI 3.98‐5.74). Long working hours (>55 hours/week) were also associated with higher odds of depression only (OR 1.42, 95% CI 1.17‐1.70), anxiety only (OR 1.63, 95% CI 1.09‐2.51), and comorbid symptoms (OR 1.64, 95% CI 1.27‐2.13). In addition, low occupational health literacy was independently associated with higher odds of all outcomes, whereas night or rotating shift work was not significantly associated with any outcome.

**Table 3.** Associations between explanatory factors and depressive, anxiety, and comorbid symptoms among participants in Chongqing, China (cross-sectional survey, January 1-December 31, 2024; N=10,112).

Variables	Depressive symptom		Anxiety symptom		Comorbid symptoms					
					Depression only		Anxiety only		Comorbidity	
	OR^a^ (95% CI)	*P* value	OR (95% CI)	*P* value	OR (95% CI)	*P* value	OR (95% CI)	*P* value	OR (95% CI)	*P* value
Sex										
Men	1.15 (1.02‐1.29)	.02	1.05 (0.89‐1.24)	.56	1.18 (1.03‐1.34)	.02	1.14 (0.83‐1.56)	.42	1.09 (0.90‐1.32)	.40
Women	Ref^b^		Ref		Ref		Ref		Ref	
Age group (years)										
16‐39	1.32 (1.16‐1.49)	<.001	1.43 (1.20‐1.71)	<.001	1.26 (1.09‐1.46)	.002	1.42 (1.03‐2.00)	.03	1.54 (1.25‐1.87)	<.001
More than 40	Ref		Ref		Ref		Ref		Ref	
Relationship status										
Single	1.39 (1.19‐1.61)	<.001	1.10 (0.89‐1.36)	.37	1.40 (1.18‐1.65)	<.001	0.81 (0.51‐1.27)	.39	1.35 (1.06‐1.70)	.01
Married	Ref		Ref		Ref		Ref		Ref	
Other	1.44 (1.14‐1.81)	.002	1.57 (1.15‐2.12)	.004	1.29 (0.98‐1.69)	.07	1.17 (0.59‐2.12)	.64	1.85 (1.30‐2.54)	<.001
Education										
Junior school and lower	Ref		Ref		Ref		Ref		Ref	
Senior middle school	1.36 (1.18‐1.56)	<.001	1.09 (0.89‐1.33)	.41	1.45 (1.22‐1.69)	<.001	1.21 (0.84‐1.76)	.31	1.20 (0.95‐1.50)	.13
Undergraduate	1.66 (1.40‐1.95)	<.001	1.44 (1.14‐1.81)	.002	1.63 (1.35‐1.98)	<.001	1.19 (0.77‐1.86)	.43	1.78 (1.37‐2.32)	<.001
Sick leave days in the past year										
None	Ref		Ref		Ref		Ref		Ref	
Less than 3 days	1.41 (1.22‐1.63)	<.001	1.48 (1.22‐1.81)	<.001	1.33 (1.13‐1.56)	<.001	1.27 (0.84‐1.90)	.25	1.70 (1.34‐2.12)	<.001
More than 3 days	2.33 (1.96‐2.78)	<.001	2.48 (2.00‐3.09)	<.001	2.11 (1.73‐2.58)	<.001	2.38 (1.50‐3.61)	<.001	3.35 (2.61‐4.29)	<.001
Weekly working hours										
35‐40 hours	Ref		Ref		Ref		Ref		Ref	
40‐55 hours	1.15 (1.00‐1.32)	.06	1.09 (0.88‐1.34)	.44	1.17 (0.99‐1.38)	.06	1.11 (0.74‐1.64)	.63	1.14 (0.90‐1.46)	.27
Over 55 hours	1.45 (1.23‐1.70)	<.001	1.50 (1.20‐1.89)	<.001	1.42 (1.17‐1.70)	<.001	1.63 (1.09‐2.51)	.02	1.64 (1.27‐2.13)	<.001
Multisite bodily pain										
No	Ref		Ref		Ref		Ref		Ref	
Less than 3 sites	2.53 (2.19‐2.91)	<.001	2.48 (1.99‐3.10)	<.001	2.48 (2.11‐2.92)	<.001	2.65 (1.76‐3.98)	<.001	3.00 (2.34‐3.91)	<.001
More than 3 sites	4.02 (3.46‐4.67)	<.001	3.91 (3.13‐4.88)	<.001	3.76 (3.18‐4.48)	<.001	3.95 (2.57‐5.95)	<.001	5.66 (4.36‐7.37)	<.001
Night shift										
Yes	1.07 (0.95‐1.21)	.27	1.00 (0.85‐1.19)	.96	1.07 (0.93‐1.22)	.36	0.91 (0.65‐1.24)	.52	1.06 (0.88‐1.28)	.53
No	Ref		Ref		Ref		Ref		Ref	
Occupational stress										
Yes	3.25 (2.89‐3.64)	<.001	3.36 (2.87‐3.94)	<.001	2.99 (2.62‐3.39)	<.001	3.51 (2.59‐4.76)	<.001	4.78 (3.98‐5.74)	<.001
No	Ref		Ref		Ref		Ref		Ref	
Occupational health literacy										
Low	1.32 (1.18‐1.49)	<.001	1.69 (1.43‐2.00)	<.001	1.25 (1.09‐1.42)	<.001	2.14 (1.55‐2.99)	<.001	1.69 (1.40‐2.05)	<.001
High	Ref		Ref		Ref		Ref		Ref	

a OR:odds ratio.

b Ref: reference.

### Variations in the Prevalence of Depressive and Anxiety Symptoms Across Industries

**Figure 1.**
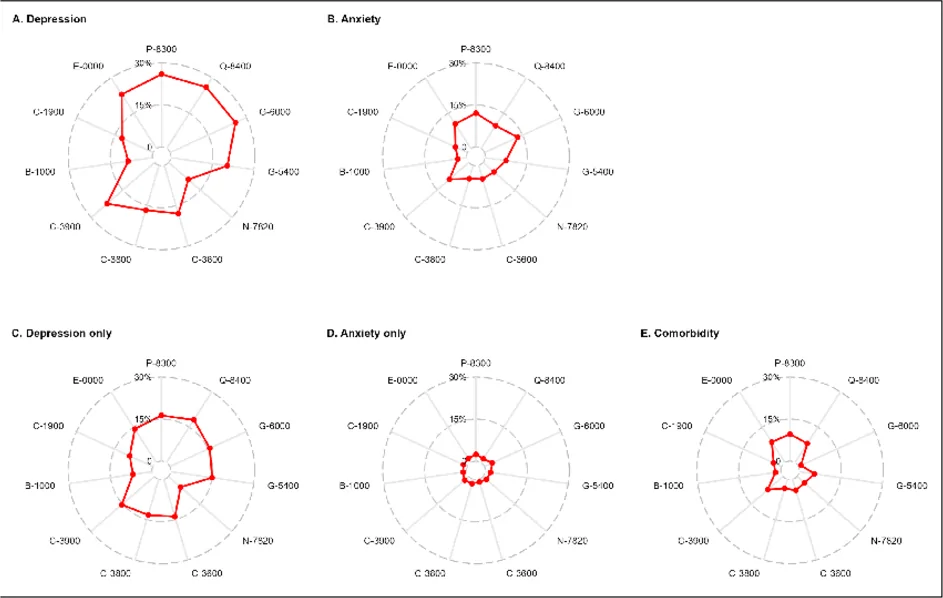
Prevalence of (A) depressive, (B) anxiety, (C) depression only, (D) anxiety only, and (E) comorbid symptoms across 11 industries in Chongqing, China (cross-sectional survey, January 1-December 31, 2024; N=10,112). C-1900: leather, fur, feather and their products, and footwear manufacturing industry; C-3600: automotive manufacturing industry; C-3800: electrical equipment manufacturing industry; E-0000: construction industry; B-1000: non-metallic mineral mining industry; C-3900: computer and communication equipment manufacturing industry; Q-8400: health care and social work industry; P-8300: education industry; N-7820: environmental sanitation industry; G-5400: transportation industry; G-6000: express delivery and food delivery industry.

Striking variations in the prevalence of depressive symptom, anxiety symptom, and comorbid symptoms across the 11 occupational groups are shown in Figure 1 and Table S2 in Multimedia Appendix 2. Depressive symptoms were most common in the health care and social work sector (26.1%, 95% CI 24.3%‐28.0%) and least common in the non-metallic mineral mining industry (8.7%, 95% CI 6.1%‐11.4%), whereas anxiety symptoms were highest among express and food delivery workers (13.1%, 95% CI 9.8%‐16.3%). Comorbid depressive-anxiety symptoms were particularly elevated in education (9.6%, 95% CI 7.5%‐11.8%), express delivery (10.0%, 95% CI 7.3%‐12.6%), and construction (8.7%, 95% CI 5.5%‐11.9%) workers, and lowest in mining (1.9%, 95% CI 1.0%‐2.8%) and leather, fur, and feather manufacturing (3.1%, 95% CI 2.7%‐3.4%). Depression only symptoms were more frequent in health care (18.1%, 95% CI 15.1%‐21.0%) and education (16.3%, 95% CI 14.3%‐18.3%), while anxiety only symptoms were relatively more common in express delivery (3.1%, 95% CI 1.8%‐4.4%).

**Figure 2.**
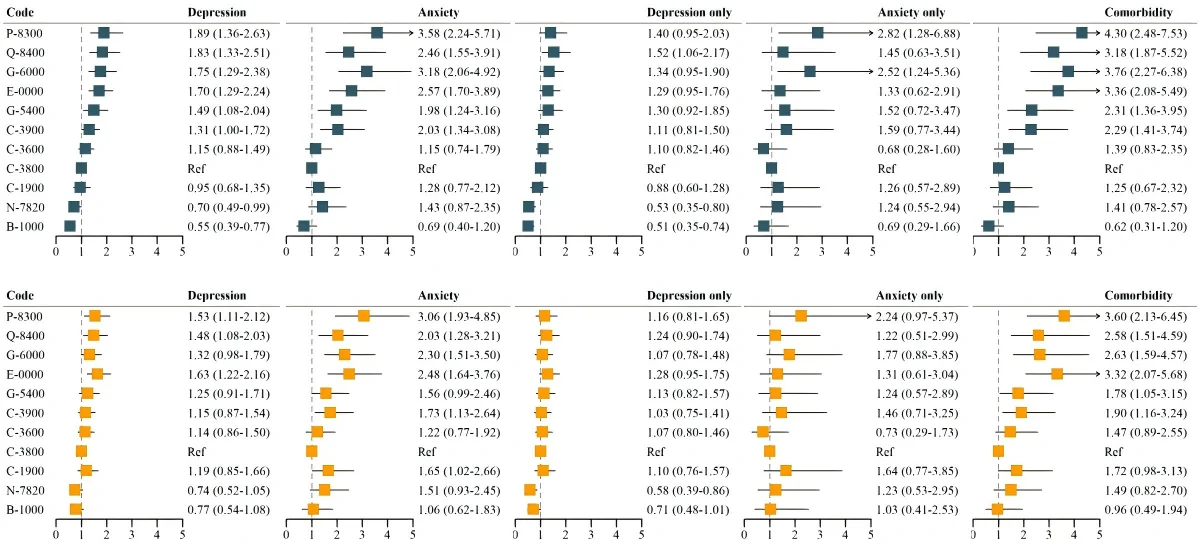
Associations between industries and depressive, anxiety, and comorbid symptoms, adjusting for covariates among participants in Chongqing, China (cross-sectional survey, January 1-December 31, 2024; N=10,112). Blue represents results adjusting for only demographic factors and yellow represents results adjusting for all covariates. C-1900: leather, fur, feather and their products, and footwear manufacturing industry; C-3600: automotive manufacturing industry; C-3800: electrical equipment manufacturing industry; E-0000: construction industry; B-1000: non-metallic mineral mining industry; C-3900: computer and communication equipment manufacturing industry; Q-8400: health care and social work industry; P-8300: education industry; N-7820: environmental sanitation industry; G-5400: transportation industry; G-6000: express delivery and food delivery industry.

Multivariable analyses reinforced differences in mental health outcomes across industries (Figure 2 and Table S3 in Multimedia Appendix 2). In models controlling for demographic factors, workers in the education, health care, express delivery, and construction industries had significantly higher odds of depression only, anxiety only, and comorbid symptoms than those in the reference industry. These associations remained after further adjustment for demographic, health, and occupational characteristics, although effect estimates were attenuated. Workers in the education, health care, express delivery, and construction industries had the highest odds of comorbid depression-anxiety symptoms among all industries. In contrast, workers in traditional production sectors, particularly non-metallic mineral mining (OR 0.68, 95% CI 0.47‐0.97), showed lower odds of such mental health symptoms, whereas the association for the leather and footwear manufacturing sector was not statistically significant (OR 1.15, 95% CI 0.81‐1.62).

Sequential adjustment analyses (Table S4 in Multimedia Appendix 2) showed different explanatory patterns across mental health outcomes. Physical health indicators markedly attenuated differences in comorbid and depressive symptoms, whereas occupational stress accounted for the largest reductions in anxiety. Demographic characteristics and night shift work explained little of the observed occupational heterogeneity.

## Discussion

### Principal Findings

In this large, multi-industry survey of Chinese workers, we identified a substantial burden of depressive symptoms, anxiety symptoms, and their comorbidity. Nearly 1 in 5 participants reported depressive symptoms, and more than 1 in 8 experienced comorbidity, with younger workers exhibiting the highest prevalence across outcomes. Occupational stress and multisite bodily pain showed the strongest associations with all mental health outcomes; long working hours, low occupational health literacy, and poorer physical health were also independently associated with higher odds of depressive symptoms, anxiety symptoms, and comorbidity. Marked heterogeneity was observed across industries: workers in education, health care, express delivery, and construction sectors had the highest prevalence and odds of adverse mental health outcomes, whereas those in traditional manufacturing and mining industries exhibited substantially lower prevalence. Collectively, these findings suggest that work-related mental health problems represent a substantial and heterogeneous public health challenge and underscore the importance of industry-specific occupational mental health strategies and supportive policy development.

The prevalence estimates in this study fall within the upper range of recent findings in China. A meta-analysis by Bin et al [3] reported pooled rates around 31% for depressive symptoms and 29% for anxiety symptoms. Globally, the prevalence of depressive symptoms in working populations from high-income countries typically ranges from 10% to 20%, and anxiety from 5% to 15%, with comorbidity often representing a more severe clinical subgroup [19]. The higher comorbidity observed among younger workers in our study echoes global evidence that early-career adults face elevated psychological distress under job insecurity, high performance demands, and limited autonomy [20]. Moreover, the industry-specific pattern we observed—particularly high burdens in education, health care, service delivery and other high-demand sectors—is consistent with prior research identifying teachers, health professionals, and service workers as high-risk groups due to emotional labor, time pressure, and intensive interpersonal demands [13]. These parallels reinforce the plausibility and generalizability of our findings and suggest shared structural determinants of work-related mental health risk across national labor contexts.

Several individual and occupational factors were independently associated with higher odds of depressive symptoms, anxiety symptoms, and their comorbidity among workers. Occupational stress showed the strongest and most consistent associations across all outcomes, consistent with the job demand-control and effort-reward imbalance models [21,22], which propose that sustained job demands combined with low control or inadequate reward may contribute to emotional exhaustion and poor mental health. Longer sick leave was also associated with higher odds of all symptom profiles, supporting previous evidence that physical and mental health are closely interconnected and may reinforce one another over time. Although working more than 55 hours per week showed relatively modest associations, this finding is consistent with accumulating evidence linking long working hours to burnout, depression symptoms, and impaired cognitive recovery. Lower occupational health literacy was independently associated with higher odds of adverse mental health outcomes, possibly because limited knowledge of occupational health and workplace protections may reduce workers’ capacity to recognize psychological symptoms, adopt effective coping strategies, and seek timely support. By contrast, night or rotating shift work was not independently associated with mental health outcomes after adjustment, suggesting that the observed associations between shift work and mental health reported in previous studies may be partly explained by related factors, such as sleep disruption, occupational stress, and other co-occurring workplace exposures, rather than shift schedules alone [23].

The pronounced industry differences observed in our study likely reflect divergent psychosocial exposures, work organization, and labor conditions. Education and health care settings are classic high emotional labor sectors where workers routinely manage intense interpersonal demands, high expectations, and mounting administrative workloads, factors linked to elevated stress and burnout in multiple reviews. These pressures, compounded by chronic resource constraints in public service settings, may contribute to sustained psychological strain [24]. Platform-based delivery workers face sustained time pressure, algorithmic management, income instability, and limited institutional protections—conditions associated with higher anxiety and depressive symptoms in recent Chinese studies of couriers and delivery riders [25]. Construction workers’ high comorbidity risk may reflect a confluence of hazardous environments, heavy physical load, injury-related pain, precarious/migrant employment, and constrained access to social and health supports [26]. In contrast, lower prevalence in some manufacturing and mining settings may be related to the more routinized tasks, stronger team cohesion, or selection/work-survival effects, although measurement and reporting differences could also be contributors. Importantly, comorbid depressive-anxiety presentations were the most disparate outcome across sectors, implying that psychosocially demanding occupations not only raise single-disorder risk but also foster more complex, clinically significant co-occurring illnesses [27,28].

Sequential adjustment analyses provided further insight into the mechanisms underlying occupational differences. For depressive-anxiety comorbidity, both physical health (eg, sick leave and multisite bodily pain) and occupational stress may contribute to severe mental health outcomes. For depression only, physical health indicators explained a substantial proportion of the between-industry variation, whereas occupational stress contributed relatively little. In contrast, for anxiety only, occupational stress consistently produced the largest attenuation across industries. Demographic characteristics and night shift work explained little of the observed occupational heterogeneity across all outcomes, highlighting workplace-related factors rather than workforce composition as the principal contributors to industry differences. These findings suggest that different mental health outcomes may arise through distinct occupational pathways, with physical health playing a greater role in depression and comorbidity, whereas psychosocial stress appears more closely linked to anxiety.

These findings carry important implications for public health practice and occupational policy. Occupational stress and physical health indicators should be prioritized as key, modifiable targets within workplace mental health strategies. Evidence-based measures—including workload redesign, stress-management interventions, and organizational policies that ensure adequate rest—may collectively mitigate psychological risk [29]. Industry-specific approaches are also essential. In high emotional labor sectors such as education and health care, interventions might focus on reducing administrative burden, improving staffing levels, and strengthening resilience and support systems [30,31]. For platform-based delivery workers, regulatory oversight of task allocation, guaranteed rest periods, and safeguards against algorithm-driven overwork are critical [32]. Among construction workers, integrating routine mental health screening, health-literacy training, and onsite counseling into occupational health services may be particularly beneficial [33]. Strengthening occupational health literacy across the workforce could further enhance early symptom recognition, help-seeking, and adoption of protective behaviors. These findings underscore the need to embed mental health more formally within China’s occupational health system and Healthy China initiatives, with emphasis on systematic monitoring and targeted prevention.

### Strengths and Limitations

This study has several strengths, including its large, multi-industry sample, the use of validated mental health instruments, and the application of mixed-effects and multinomial models that allowed examination of both individual-level determinants and industry-level heterogeneity. However, several limitations should be noted. The cross-sectional design precludes causal inference, and reliance on self-reported symptoms introduces the possibility of reporting bias. This study was conducted in a single province-level municipality, Chongqing, one of China’s largest municipalities that has undergone rapid industrialization, urbanization, and labor-force diversification. These characteristics make it an informative setting for investigating occupational mental health in rapidly developing urban environments. Nevertheless, caution is warranted when generalizing the findings to other regions, and future studies including multiple provinces and cities are needed. Although marital status was adjusted for, other family-related factors, including family support, work-family conflict, and caregiving responsibilities, were not measured. Future studies should examine how occupational and family environments jointly influence workers’ mental health. Finally, important industry-specific psychosocial constructs—such as job control, organizational justice, and supervisor support—were not measured and should be incorporated into future research to improve explanatory depth and intervention relevance.

### Conclusion

In conclusion, this study indicates substantial and heterogeneous burdens of depressive, anxiety, and comorbid symptoms among workers across multiple industries in China. The findings identify key modifiable risk factors and high-risk sectors that require policy attention. Integrating mental health promotion into occupational health systems and tailoring interventions to sector-specific needs will be critical for reducing work-related psychological burdens and advancing national mental health goals.

## Supplementary material

10.2196/91782 Multimedia Appendix 1 Study design details.

10.2196/91782 Multimedia Appendix 2 Additional tables on the prevalence of depressive symptoms, anxiety symptoms, and comorbid symptoms, and their associations with industries.

10.2196/91782 Checklist 1 STROBE checklist.

## Abbreviations

GAD-7: 7-item Generalized Anxiety Disorder scale
OR: odds ratio
PHQ-9: 9-item Patient Health Questionnaire
STROBE: Strengthening the Reporting of Observational Studies in Epidemiology
