# Supplementary material for: Depressive and Anxiety Symptoms and Their Comorbidity Across Industries: Multi-Industry Cross-Sectional Study in China
Source: JMIR Public Health Surveill. 2026 Aug 21;12:e91782. doi: 10.2196/91782 (PMC13495997; doi:10.2196/91782)
Supplement: Supplementary file 1 [file publichealth-v12-e91782-s001.docx]

**Survey Development**

This survey was conducted from January 1 to December 31, 2024, in Chongqing, China, as part of the Occupational Health Literacy Surveillance Program. The surveillance aimed to monitor occupational health literacy and related mental health outcomes among occupational populations, identify factors associated with occupational health literacy and mental health, evaluate the effectiveness of occupational health promotion initiatives, and provide evidence to inform occupational health policies and targeted intervention strategies.

The survey was developed in accordance with the National Occupational Health Literacy Surveillance Protocol. Based on the Industrial Classification for National Economic Activities (GB/T 4754-2017), the current protocol designated six priority industries in the secondary sector for surveillance, including leather, fur, feather and footwear manufacturing (C-1900), automotive manufacturing (C-3600), electrical equipment manufacturing (C-3800), and construction (E-0000), non-metallic mineral mining (B-1000) and computer, communication, and other electronic equipment manufacturing (C-3900). Five industries in the tertiary sector were selected, including health care and social work (Q-8400), education (P-8300), environmental sanitation (N-7820), transportation (G-5400), and express and food delivery services (G-6000). These 11 industries represent major occupational groups with diverse workplace environments and psychosocial exposures across Chongqing.

**Participant Recruitment**

Eligible participants were front-line workers aged 16-59 years who had been employed in their current occupation for at least six months. Front-line workers in the secondary sector included production and manufacturing workers directly engaged in industrial operations, whereas administrative staff were excluded. In the tertiary sector, eligible participants comprised physicians, nurses, teachers, environmental sanitation workers, drivers employed by taxi, bus, freight, or ride-hailing companies, and express and food-delivery workers.

Participant recruitment required a minimum of 800 completed questionnaires per industry. To accommodate exclusions and to ensure adequate statistical power, approximately 10,200 workers were targeted for recruitment. A multistage stratified cluster sampling design was employed. For the secondary sector, enterprises served as the primary sampling units and were stratified by enterprise size (large, medium, and small/micro). Enterprises within each stratum were randomly selected, with target sample allocations of approximately 160, 240, and 400 workers, respectively. Large enterprises accounted for no more than 10% of all sampled enterprises to improve representativeness across enterprise sizes. For the tertiary sector, five districts (YZ, BN, KZ, JLP, and YC) were selected. Within each district, institutions were randomly sampled according to industry-specific sampling schemes. Specifically, healthcare workers were recruited from 1-2 secondary or tertiary hospitals and 2-3 primary healthcare institutions; teachers from 1-3 primary schools and 1-3 secondary schools; sanitation workers from 1-3 environmental sanitation agencies; transportation workers from 2-3 taxi, public transport, freight, or ride-hailing companies; and delivery workers from 1-3 courier or food-delivery companies. Each district aimed to recruit at least 200 participants from each service-sector industry (**Table A**).

All eligible workers were invited to complete a standardized, anonymous, self-administered electronic questionnaire after providing written informed consent.

**Data weighting**

To improve the representativeness of prevalence estimates, post-stratification weights were derived using the employed population of Chongqing from the Seventh National Population Census (2020). The reference population was stratified by sex and 5-year age groups among employed individuals aged 16-59 years (**Table B**). Weighting factors were calculated as the ratio of the census population to the survey sample within each age-sex stratum and applied to all prevalence estimates. Standardized prevalence estimates and 95% confidence intervals were calculated using these weights, with variances estimated by Taylor series linearization.

Occupation-specific census data jointly stratified by age and sex were unavailable; therefore, post-stratification was based on the age-sex distribution of the overall employed population rather than that of individual occupations. Accordingly, the resulting weights improve the representativeness of the overall working population but should not be interpreted as providing fully occupation-specific population estimates.

**Table A.** Distribution of sampled industries and participants across districts/counties in Chongqing, China (cross-sectional survey, January 1-December 31, 2024; N=10112).

| ID | District | Sample size | Cluster Code | P-8300 | Q-8400 | G-6000 | G-5400 | N-7820 | C-3600 | C-3800 | C-3900 | B-1000 | C-1900 | E-0000 |
| --- | --- | --- | --- | --- | --- | --- | --- | --- | --- | --- | --- | --- | --- | --- |
| 1 | BN | 1186 | 1 | 220 | 232 | 260 | 223 | 219 | – | 32 | – | – | – | – |
| 2 | DJ | 118 | 2 | – | – | – | – | – | – | 31 | 195 | 23 | – | – |
| 3 | LP | 32 |  |  |  |  |  |  |  |  |  |  |  |  |
| 4 | ZX | 99 |  |  |  |  |  |  |  |  |  |  |  |  |
| 5 | DDK | 260 | 3 | – | – | – | – | – | – | 91 | 106 | – | 63 | – |
| 6 | CK | 38 | 4 | – | – | – | – | – | – | – | – | 169 | – | 139 |
| 7 | FJ | 111 |  |  |  |  |  |  |  |  |  |  |  |  |
| 8 | WS | 149 |  |  |  |  |  |  |  |  |  |  |  |  |
| 9 | YY | 10 |  |  |  |  |  |  |  |  |  |  |  |  |
| 10 | CS | 200 | 5 | – | – | – | – | – | 57 | 214 | 89 | 24 |  | 62 |
| 11 | PL | 222 |  |  |  |  |  |  |  |  |  |  |  |  |
| 12 | WX | 24 |  |  |  |  |  |  |  |  |  |  |  |  |
| 13 | GX | 229 | 6 | – | – | – | – | – | 100 | 129 | – | – | – | – |
| 14 | JB | 230 | 7 | – | – | – | – | – | 63 | 88 | – | – | 79 | – |
| 15 | JLP | 1378 | 8 | 242 | 252 | 212 | 210 | 238 | 97 | – | – | – | – | 127 |
| 16 | KZ | 1114 | 9 | 245 | 234 | 205 | 219 | 211 | – |  | – | – | – | – |
| 17 | LJ | 284 | 10 | – | – | – | – | – | 209 | 75 | – | – | – | – |
| 18 | TL | 250 | 11 | – | – | – | – | – | – | – | 110 |  | 140 |  |
| 19 | WZ | 286 | 12 | – | – | – | – | – | 91 | 157 | – | 10 | – | 28 |
| 20 | NA | 28 | 13 | 239 | 223 | 240 | 214 | 220 | – | – | – | – | – | 28 |
| 21 | YZ | 1136 |  |  |  |  |  |  |  |  |  |  |  |  |
| 22 | BS | 572 | 14 | – | – | – | – | – | – | – | – | – | 572 | – |
| 23 | QJ | 152 | 15 | – | – | – | – | – | 50 | – | – | – | – | 187 |
| 24 | WSJ | 85 |  |  |  |  |  |  |  |  |  |  |  |  |
| 25 | BB | 301 | 16 | – | – | – | – | – | 195 | 49 | 252 | – | – | – |
| 26 | SPB | 96 |  |  |  |  |  |  |  |  |  |  |  |  |
| 27 | YB | 99 |  |  |  |  |  |  |  |  |  |  |  |  |
| 28 | DZ | 57 | 17 | – | – | – | – | – | 57 | – | – | – | 30 | 141 |
| 29 | RC | 96 |  |  |  |  |  |  |  |  |  |  |  |  |
| 30 | TN | 75 |  |  |  |  |  |  |  |  |  |  |  |  |
| 31 | HC | 253 | 18 | – | – | – | – | – | – | – | 124 | 101 | – | 28 |
| 32 | JJ | 110 | 19 | – | – | – | – | – | – | 30 | 45 | 56 | 69 | 63 |
| 33 | YC | 153 |  |  |  |  |  |  |  |  |  |  |  |  |
| 34 | NC | 31 | 20 | – | – | – | – | – | – | – | – | 184 | – | 65 |
| 35 | WL | 34 |  |  |  |  |  |  |  |  |  |  |  |  |
| 36 | FD | 184 |  |  |  |  |  |  |  |  |  |  |  |  |
| 37 | SZT | 79 | 21 | – | – | – | – | – | – | 37 | – | 323 | – | 70 |
| 38 | SZ | 37 |  |  |  |  |  |  |  |  |  |  |  |  |
| 39 | XST | 31 |  |  |  |  |  |  |  |  |  |  |  |  |
| 40 | XS | 37 |  |  |  |  |  |  |  |  |  |  |  |  |
| 41 | YYT | 24 |  |  |  |  |  |  |  |  |  |  |  |  |
| 42 | YY | 33 |  |  |  |  |  |  |  |  |  |  |  |  |
| 43 | PSM | 78 |  |  |  |  |  |  |  |  |  |  |  |  |
| 44 | QJ | 111 |  |  |  |  |  |  |  |  |  |  |  |  |

**Table B.** Calculation of post-stratification weightsin Chongqing, China (cross-sectional survey, January 1-December 31, 2024; N=10112)..

| Sex | Age_group | Sample size | Population number |
| --- | --- | --- | --- |
| Male | 16-19 | 67 | 8575 |
| Male | 20-24 | 258 | 53919 |
| Male | 25-29 | 592 | 97368 |
| Male | 30-34 | 760 | 118828 |
| Male | 35-39 | 881 | 86920 |
| Male | 40-44 | 668 | 74485 |
| Male | 45-49 | 644 | 143800 |
| Male | 50-54 | 857 | 129114 |
| Male | 55-60 | 639 | 86317 |
| Female | 16-19 | 34 | 4957 |
| Female | 20-24 | 158 | 43246 |
| Female | 25-29 | 606 | 75296 |
| Female | 30-34 | 712 | 89693 |
| Female | 35-39 | 889 | 67616 |
| Female | 40-44 | 708 | 60295 |
| Female | 45-49 | 697 | 110601 |
| Female | 50-54 | 580 | 78962 |
| Female | 55-60 | 362 | 43483 |
