# Supplementary material for: Depressive and Anxiety Symptoms and Their Comorbidity Across Industries: Multi-Industry Cross-Sectional Study in China
Source: JMIR Public Health Surveill. 2026 Aug 21;12:e91782. doi: 10.2196/91782 (PMC13495997; doi:10.2196/91782)
Supplement: Supplementary file 2 [file publichealth-v12-e91782-s002.docx]

**Table S1.** Crude prevalence of depressive symptom, anxiety symptom, and comorbid symptoms among participants in Chongqing, China (cross-sectional survey, January 1-December 31, 2024; N=10112), overall and by subgroups.

|  | Depressive symptom | Anxiety symptom | Comobid symptoms | | |
| --- | --- | --- | --- | --- | --- |
|  |  |  | Depression only | Anxiety only | Comorbidity |
| Overall | 18.9 (16.5–21.3) | 7.8 (6.3–9.2) | 12.9 (11.6–14.3) | 1.8 (1.5–2.1) | 6.0 (4.8–7.2) |
| Sex |  |  |  |  |  |
| Men | 19.5 (16.9–22.1) | 7.8 (6.3–9.3) | 13.6 (11.8–15.3) | 1.8 (1.4–2.3) | 6.0 (4.8–7.1) |
| Women | 18.2 (15.5–21.0) | 7.7 (6.0–9.4) | 12.2 (10.9–13.6) | 1.7 (1.4–2.0) | 6.0 (4.4–7.6) |
| Age group |  |  |  |  |  |
| 16-39 | 23.1 (20.7–25.4) | 9.6 (7.7–11.5) | 15.5 (14.1–16.8) | 2.0 (1.6–2.4) | 7.6 (6.0–9.2) |
| 40-59 | 14.9 (12.4–17.5) | 6.0 (4.7–7.3) | 10.5 (9.0–11.9) | 1.6 (1.2–1.9) | 4.4 (3.2–5.7) |
| Relationship status |  |  |  |  |  |
| Single | 26.6 (24.2–29.0) | 9.9 (8.1–11.6) | 18.3 (16.4–20.3) | 1.6 (1.1–2.1) | 8.3 (6.7–9.8) |
| Married | 17.0 (14.7–19.2) | 7.1 (5.7–8.5) | 11.7 (10.4–12.9) | 1.8 (1.5–2.1) | 5.3 (4.1–6.4) |
| Others | 23.5 (19.0–27.9) | 11.3 (7.1–15.4) | 14.3 (10.7–17.9) | 2.1 (0.8–3.3) | 9.2 (5.2–13.1) |
| Education |  |  |  |  |  |
| Junior middle school and low | 13.8 (12.0–15.7) | 6.2 (5.0–7.4) | 9.3 (8.2–10.4) | 1.7 (1.4–2.0) | 4.5 (3.5–5.5) |
| Senior middle school | 20.0 (17.5–22.5) | 7.5 (6.2–8.9) | 14.3 (12.4–16.3) | 1.9 (1.4–2.3) | 5.7 (4.6–6.7) |
| Undergraduate | 25.2 (23.2–27.1) | 10.6 (8.5–12.7) | 16.4 (15.2–17.6) | 1.8 (1.0–2.7) | 8.8 (7.2–10.4) |
| Sick leave days last year |  |  |  |  |  |
| None | 15.4 (13.0–17.7) | 5.8 (4.5–7.1) | 11.0 (9.6–12.4) | 1.5 (1.2–1.7) | 4.3 (3.2–5.4) |
| Less than 3 days | 28.0 (24.9–31.1) | 12.2 (9.4–15.0) | 18.1 (15.7–20.6) | 2.3 (1.3–3.3) | 9.9 (7.6–12.1) |
| More than 3 days | 38.8 (35.6–42.0) | 19.5 (16.1–23.0) | 23.0 (20.4–25.4) | 3.7 (2.4–5.1) | 15.8 (12.6–18.9) |
| Weekly working hours |  |  |  |  |  |
| 35-40 hours | 15.6 (13.1–18.2) | 6.3 (4.9–7.7) | 10.8 (9.0–12.7) | 1.5 (1.0–2.1) | 4.8 (3.6–6.0) |
| 40-55 hours | 17.9 (14.9–20.9) | 7.0 (5.3–8.6) | 12.5 (10.8–14.2) | 1.6 (1.3–1.8) | 5.4 (4.0–6.9) |
| Over 55 hours | 24.4 (21.2–27.5) | 10.8 (7.7–14.0) | 16.0 (14.0–18.1) | 2.5 (1.6–3.4) | 8.4 (6.0–10.9) |
| The count of painful body sites | |  |  |  |  |
| No | 8.6 (7.3–9.8) | 2.9 (2.3–3.6) | 6.5 (5.5–7.5) | 0.9 (0.6–1.2) | 2.1 (1.5–2.6) |
| Less than 3 | 21.7 (19.3–24.1) | 8.5 (7.1–10.0) | 15.3 (13.5–17.0) | 2.1 (1.6–2.6) | 6.4 (5.3–7.5) |
| More than 3 | 35.4 (32.7–38.0) | 16.2 (14.2–18.2) | 22.3 (20.3–24.2) | 3.1 (2.6–3.7) | 13.1 (11.2–15.0) |
| Night Shift |  |  |  |  |  |
| Yes | 21.7 (19.2–24.1) | 9.1 (7.7–10.5) | 14.7 (12.8–16.7) | 2.2 (1.7–2.7) | 6.9 (5.9–8.0) |
| No | 16.6 (13.9–19.4) | 6.7 (5.0–8.3) | 11.4 (9.9–13.0) | 1.5 (1.2–1.8) | 5.2 (3.7–6.6) |
| Occupational stress |  |  |  |  |  |
| Yes | 37.2 (33.9–40.6) | 17.7 (15.2–20.3) | 23.1 (21.0–25.3) | 3.6 (3.0–4.3) | 3.3 (2.6–4.1) |
| No | 13.0 (11.2–14.7) | 4.5 (3.6–5.5) | 9.6 (8.5–10.7) | 1.2 (0.8–1.5) | 14.1 (11.6–16.6) |
| Occupational health literacy |  |  |  |  |  |
| Low | 21.6 (19.3–23.8) | 10.0 (8.8–11.2) | 14.0 (12.5–15.6) | 2.4 (2.1–2.8) | 7.5 (6.5–8.5) |
| High | 16.2 (13.2–19.1) | 5.5 (3.6–7.3) | 11.8 (10.1–13.6) | 1.1 (0.7–1.5) | 4.4 (2.8–5.9) |

**Table S2.** Prevalence of depressive symptom, anxiety symptom, and comorbid symptoms among participants in Chongqing, China (cross-sectional survey, January 1-December 31, 2024; N=10112), stratified by industries.

| Industry  Categories | Depressive symptom | Anxiety symptom | Comobid symptoms | | |
| --- | --- | --- | --- | --- | --- |
|  |  |  | Depression only | Anxiety only | Comorbidity |
| Crude prevalence | |  |  |  |  |
| P–8300 | 26.1 (22.1–30.1) | 12.9 (10.3–15.5) | 15.8 (12.8–18.7) | 2.5 (1.2–3.9) | 10.4 (8.3–12.4) |
| Q–8400 | 26.5 (24.1–28.8) | 9.9 (7.5–12.3) | 18.1 (15.3–20.8) | 1.5 (0.8–2.2) | 8.4 (6.1–10.7) |
| G–6000 | 26.1 (22.9–29.2) | 13.0 (9.4–16.5) | 16.1 (14.2–18.0) | 3.1 (1.8–4.3) | 9.9 (6.9–13.0) |
| G–5400 | 20.8 (19.7–21.8) | 7.7 (6.6–8.8) | 15.0 (14.0–16.1) | 2.0 (1.1–2.8) | 5.8 (4.8–6.8) |
| N–7820 | 9.2 (4.5–14.0) | 5.1 (2.4–7.7) | 5.7 (2.2–9.3) | 1.6 (0.2–3.0) | 3.5 (1.7–5.3) |
| C–3600 | 18.2 (14.9–21.4) | 5.2 (3.8–6.6) | 13.9 (11.1–16.7) | 1.0 (0.5–1.5) | 4.2 (3.0–5.5) |
| C–3800 | 15.5 (11.5–19.6) | 4.4 (2.7–6.1) | 12.5 (9.0–16.1) | 1.4 (0.7–2.0) | 3.0 (1.8–4.2) |
| C–3900 | 20.7 (15.2–26.3) | 8.8 (6.8–10.8) | 14.0 (10.3–17.7) | 2.1 (1.3–2.9) | 6.7 (4.4–9.1) |
| B–1000 | 8.8 (6.1–11.5) | 2.9 (1.8–4.0) | 7.0 (4.6–9.3) | 1.1 (0.2–2.1) | 1.8 (0.9–2.7) |
| C–1900 | 12.5 (10.4–14.6) | 4.8 (4.2–5.4) | 9.3 (7.5–11.2) | 1.7 (1.3–2.1) | 3.1 (2.9–3.4) |
| E–0000 | 23.0 (16.5–29.5) | 10.3 (6.3–14.4) | 14.4 (10.7–18.1) | 1.7 (0.7–2.7) | 8.6 (5.2–12.0) |
| Weighted prevalence | |  |  |  |  |
| P–8300 | 26.0 (22.9–29.1) | 12.1 (9.4–14.7) | 16.3 (14.3–18.3) | 2.4 (1.2–3.6) | 9.6 (7.5–11.8) |
| Q–8400 | 26.1 (24.3–28.0) | 9.7 (7.4–12.0) | 18.1 (15.1–21.0) | 1.6 (0.9–2.4) | 8.1 (5.7–10.4) |
| G–6000 | 25.7 (23.1–28.3) | 13.1 (9.8–16.3) | 15.7 (13.5–18.0) | 3.1 (1.8–4.4) | 10.0 (7.3–12.6) |
| G–5400 | 20.4 (19.7–21.2) | 7.5 (6.1–8.9) | 15.0 (14.2–15.7) | 2.0 (1.1–3.0) | 5.5 (4.7–6.2) |
| N–7820 | 9.3 (4.8–13.8) | 5.2 (2.6–7.7) | 5.8 (2.5–9.1) | 1.6 (0.1–3.2) | 3.5 (2.0–5.0) |
| C–3600 | 18.0 (14.9–21.0) | 5.1 (3.7–6.5) | 13.9 (11.0–16.8) | 1.0 (0.4–1.5) | 4.1 (3.0–5.3) |
| C–3800 | 16.7 (12.5–20.9) | 5.0 (3.3–6.7) | 13.3 (9.6–17.0) | 1.6 (0.9–2.2) | 3.4 (2.0–4.8) |
| C–3900 | 22.5 (16.1–29.0) | 9.2 (6.6–11.8) | 15.4 (11.5–19.3) | 2.0 (1.3–2.8) | 7.2 (4.1–10.2) |
| B–1000 | 8.7 (6.1–11.4) | 3.2 (2.3–4.0) | 6.9 (4.4–9.3) | 1.3 (0.3–2.3) | 1.9 (1.0–2.8) |
| C–1900 | 12.2 (10.0–14.4) | 4.7 (4.1–5.2) | 9.1 (7.1–11.1) | 1.6 (1.1–2.0) | 3.1 (2.7–3.4) |
| E–0000 | 23.0 (16.7–29.3) | 10.4 (6.5–14.4) | 14.2 (10.7–17.8) | 1.7 (0.6–2.9) | 8.7 (5.5–11.9) |

Note: C–1900: leather, fur, feather and their products, and footwear manufacturing industry; C–3600: automotive manufacturing industry; C–3800: electrical equipment manufacturing industry; E–0000: construction industry; B–1000: non–metallic mineral mining industry; C–3900: computer and communication equipment manufacturing industry; Q–8400: health care and social work industry; P–8300: education industry; N–7820: environmental sanitation industry; G–5400: transportation industry; G–6000: express delivery and food delivery industry.

**Table S3.** Associations between industries and depressive symptom, anxiety symptom, and comorbid symptoms adjusting for covariates among participants in Chongqing, China (cross-sectional survey, January 1-December 31, 2024; N=10112).

|  | Depressive symptom | | Anxiety symptom | | Comobid symptoms | | | | | |
| --- | --- | --- | --- | --- | --- | --- | --- | --- | --- | --- |
|  |  |  |  |  | Depression only |  | Anxiety only |  | Comorbidity |  |
|  | OR (95%CI) | *p* | OR (95%CI) | *p* | OR (95%CI) | *p* | OR (95%CI) | *p* | OR (95%CI) | *p* |
| **Model 1: demographic adjusted** | | |  |  |  |  |  |  |  |  |
| P-8300 | 1.89 (1.36–2.63) | <0.001 | 3.58 (2.24–5.71) | <0.001 | 1.40 (0.95–2.03) | 0.08 | 2.82 (1.28–6.88) | 0.01 | 4.30 (2.48–7.53) | <0.001 |
| Q-8400 | 1.83 (1.33–2.51) | <0.001 | 2.46 (1.55–3.91) | <0.001 | 1.52 (1.06–2.17) | 0.02 | 1.45 (0.63–3.51) | 0.38 | 3.18 (1.87–5.52) | <0.001 |
| G-6000 | 1.75 (1.29–2.38) | <0.001 | 3.18 (2.06–4.92) | <0.001 | 1.34 (0.95–1.90) | 0.09 | 2.52 (1.24–5.36) | 0.01 | 3.76 (2.27–6.38) | <0.001 |
| G-5400 | 1.49 (1.08–2.04) | 0.01 | 1.98 (1.24–3.16) | 0.004 | 1.30 (0.92–1.85) | 0.14 | 1.52 (0.72–3.47) | 0.28 | 2.31 (1.36–3.95) | 0.002 |
| N-7820 | 0.70 (0.49–0.99) | 0.04 | 1.43 (0.87–2.35) | 0.16 | 0.53 (0.35–0.80) | 0.002 | 1.24 (0.55–2.94) | 0.62 | 1.41 (0.78–2.57) | 0.25 |
| C-3600 | 1.15 (0.88–1.49) | 0.29 | 1.15 (0.74–1.79) | 0.55 | 1.10 (0.82–1.46) | 0.53 | 0.68 (0.28–1.60) | 0.40 | 1.39 (0.83–2.35) | 0.200 |
| C-3800 | Ref |  | Ref |  | Ref |  | Ref |  | Ref |  |
| C-3900 | 1.31 (1.00–1.72) | 0.04 | 2.03 (1.34–3.08) | <0.001 | 1.11 (0.81–1.50) | 0.51 | 1.59 (0.77–3.44) | 0.21 | 2.29 (1.41–3.74) | <0.001 |
| B-1000 | 0.55 (0.39–0.77) | <0.001 | 0.69 (0.40–1.20) | 0.19 | 0.51 (0.35–0.74) | <0.001 | 0.69 (0.29–1.66) | 0.41 | 0.62 (0.31–1.20) | 0.15 |
| C-1900 | 0.95 (0.68–1.35) | 0.79 | 1.28 (0.77–2.12) | 0.34 | 0.88 (0.60–1.28) | 0.50 | 1.26 (0.57–2.89) | 0.57 | 1.25 (0.67–2.32) | 0.48 |
| E-0000 | 1.70 (1.29–2.24) | <0.001 | 2.57 (1.70–3.89) | <0.001 | 1.29 (0.95–1.76) | 0.11 | 1.33 (0.62–2.91) | 0.47 | 3.36 (2.08–5.49) | <0.001 |
| **Model 2: full adjusted** | |  |  |  |  |  |  |  |  |  |
| P-8300 | 1.53 (1.11–2.12) | 0.010 | 3.06 (1.93–4.85) | <0.001 | 1.16 (0.81–1.65) | 0.403 | 2.24 (0.97–5.37) | 0.058 | 3.60 (2.13–6.45) | <0.001 |
| Q-8400 | 1.48 (1.08–2.03) | 0.016 | 2.03 (1.28–3.21) | 0.002 | 1.24 (0.90–1.74) | 0.186 | 1.22 (0.51–2.99) | 0.663 | 2.58 (1.51–4.59) | 0.001 |
| G-6000 | 1.32 (0.98–1.79) | 0.069 | 2.30 (1.51–3.50) | <0.001 | 1.07 (0.78–1.48) | 0.669 | 1.77 (0.88–3.85) | 0.110 | 2.63 (1.59–4.57) | <0.001 |
| G-5400 | 1.25 (0.91–1.71) | 0.164 | 1.56 (0.99–2.46) | 0.056 | 1.13 (0.82–1.57) | 0.462 | 1.24 (0.57–2.89) | 0.616 | 1.78 (1.05–3.15) | 0.034 |
| N-7820 | 0.74 (0.52–1.05) | 0.095 | 1.51 (0.93–2.45) | 0.093 | 0.58 (0.39–0.86) | 0.009 | 1.23 (0.53–2.95) | 0.606 | 1.49 (0.82–2.70) | 0.192 |
| C-3600 | 1.14 (0.86–1.50) | 0.363 | 1.22 (0.77–1.92) | 0.398 | 1.07 (0.80–1.46) | 0.644 | 0.73 (0.29–1.73) | 0.463 | 1.47 (0.89–2.55) | 0.143 |
| C-3800 | Ref |  | Ref |  | Ref |  | Ref |  | Ref |  |
| C-3900 | 1.15 (0.87–1.54) | 0.324 | 1.73 (1.13–2.64) | 0.011 | 1.03 (0.75–1.41) | 0.826 | 1.46 (0.71–3.25) | 0.315 | 1.90 (1.16–3.24) | 0.008 |
| B-1000 | 0.77 (0.54–1.08) | 0.132 | 1.06 (0.62–1.83) | 0.827 | 0.71 (0.48–1.01) | 0.058 | 1.03 (0.41–2.53) | 0.944 | 0.96 (0.49–1.94) | 0.905 |
| C-1900 | 1.19 (0.85–1.66) | 0.315 | 1.65 (1.02–2.66) | 0.042 | 1.10 (0.76–1.57) | 0.617 | 1.64 (0.77–3.85) | 0.204 | 1.72 (0.98–3.13) | 0.056 |
| E-0000 | 1.63 (1.22–2.16) | <0.001 | 2.48 (1.64–3.76) | <0.001 | 1.28 (0.95–1.75) | 0.108 | 1.31 (0.61–3.04) | 0.469 | 3.32 (2.07–5.68) | <0.001 |

Note: model 1 adjusted for only demographic factors, and model 2 adjusted for all covariates.

C-1900: leather, fur, feather and their products, and footwear manufacturing industry; C-3600: automotive manufacturing industry; C-3800: electrical equipment manufacturing industry; E-0000: construction industry; B-1000: non-metallic mineral mining industry; C-3900: computer and communication equipment manufacturing industry; Q-8400: health care and social work industry; P-8300: education industry; N-7820: environmental sanitation industry; G-5400: transportation industry; G-6000: express delivery and food delivery industry.

**Table S4.** Sequentially adjusted associations between industry and mental health outcomes showing the contribution of demographic, health, and occupational factors in Chongqing, China (cross-sectional survey, January 1-December 31, 2024; N=10112).

| **Industries** | **Model 1**: no adjustment | **Model 2:** adjusting for age, sex, education, and marital | **Model 3:** adding sick leave and multisite bodily aches | **Model 4**: adding working hours | **Model 5**: adding occupational stress | **Model 6**: adding occupational health literacy | **Model 7**: adding night shift |
| --- | --- | --- | --- | --- | --- | --- | --- |
| **Cormobidity** |  |  |  |  |  |  |  |
| P-8300 | 4.32 (2.63–7.39)^**^ | 4.30 (2.48–7.53)^**^ | 3.12 (1.86–5.50)^**^ | 3.14 (1.87–5.44)^**^ | 3.71 (2.21–6.49)^**^ | 3.53 (2.07–6.25)^**^ | 3.60 (2.13–6.45)^**^ |
| Q-8400 | 3.49 (2.10–5.98)^**^ | 3.18 (1.87–5.52)^**^ | 2.70 (1.61–4.67)^**^ | 2.75 (1.66–4.79)^**^ | 2.56 (1.51–4.48)^**^ | 2.63 (1.57–4.61)^**^ | 2.58 (1.51–4.59)^**^ |
| G-6000 | 4.18 (2.56–7.09)^**^ | 3.76 (2.27–6.38)^**^ | 3.60 (2.25–6.03)^**^ | 3.32 (2.04–5.59)^**^ | 2.97 (1.85–4.97)^**^ | 2.72 (1.68–4.64)^**^ | 2.63 (1.59–4.57)^**^ |
| E-0000 | 3.42 (2.14–5.59)^**^ | 3.36 (2.08–5.49)^**^ | 3.50 (2.20–5.78)^**^ | 3.51 (2.22–5.80)^**^ | 3.45 (2.15–5.68)^**^ | 3.36 (2.09–5.62)^**^ | 3.32 (2.07–5.68)^**^ |
| G-5400 | 2.23 (1.29–3.90)^**^ | 2.31 (1.36–3.95)^**^ | 2.04 (1.22–3.54)^**^ | 1.98 (1.18–3.46)^**^ | 1.90 (1.14–3.32)^*^ | 1.81 (1.06–3.19)^*^ | 1.78 (1.05–3.15)^*^ |
| C-3900 | 2.50 (1.57–4.13)^**^ | 2.29 (1.41–3.74)^**^ | 2.45 (1.50–4.14)^**^ | 2.22 (1.36–3.74)^**^ | 2.01 (1.22–3.34)^**^ | 1.99 (1.23–3.38)^**^ | 1.90 (1.16–3.24)^**^ |
| C-3600 | 1.40 (0.84–2.35) | 1.39 (0.83–2.35) | 1.37 (0.82–2.32) | 1.38 (0.82–2.36) | 1.38 (0.81–2.33) | 1.47 (0.87–2.52) | 1.47 (0.89–2.55) |
| C-1900 | 1.09 (0.59–2.04) | 1.25 (0.67–2.32) | 1.43 (0.80–2.63) | 1.47 (0.82–2.68) | 1.63 (0.91–2.96) | 1.69 (0.94–3.14) | 1.72 (0.98–3.13) |
| N-7820 | 1.16 (0.65–2.08) | 1.41 (0.78–2.57) | 1.62 (0.93–2.92) | 1.63 (0.91–2.98) | 1.61 (0.92–2.91) | 1.46 (0.82–2.63) | 1.49 (0.82–2.70) |
| B-1000 | 0.60 (0.30–1.16) | 0.62 (0.31–1.20) | 0.80 (0.40–1.53) | 0.79 (0.40–1.55) | 0.92 (0.47–1.77) | 0.96 (0.49–1.88) | 0.96 (0.49–1.94) |
| **Depression only** |  |  |  |  |  |  |  |
| P-8300 | 1.49 (1.05–2.10)^*^ | 1.40 (0.95–2.03) | 1.10 (0.76–1.59) | 1.09 (0.76–1.58) | 1.18 (0.82–1.69) | 1.14 (0.79–1.66) | 1.16 (0.81–1.65) |
| Q-8400 | 1.68 (1.20–2.38)^**^ | 1.52 (1.06–2.17)^*^ | 1.33 (0.95–1.89) | 1.33 (0.95–1.89) | 1.25 (0.90–1.77) | 1.25 (0.89–1.78) | 1.24 (0.90–1.74) |
| G-6000 | 1.52 (1.07–2.15)^*^ | 1.34 (0.95–1.90) | 1.35 (0.96–1.88) | 1.26 (0.91–1.78) | 1.16 (0.83–1.62) | 1.10 (0.79–1.52) | 1.07 (0.78–1.48) |
| E-0000 | 1.37 (1.00–1.87) | 1.29 (0.95–1.76) | 1.35 (0.99–1.85) | 1.33 (0.98–1.82) | 1.31 (0.96–1.80) | 1.27 (0.94–1.74) | 1.28 (0.95–1.75) |
| G-5400 | 1.30 (0.92–1.85) | 1.30 (0.92–1.85) | 1.25 (0.88–1.75) | 1.19 (0.86–1.70) | 1.17 (0.83–1.64) | 1.13 (0.81–1.58) | 1.13 (0.82–1.57) |
| C-3900 | 1.17 (0.86–1.59) | 1.11 (0.81–1.50) | 1.24 (0.91–1.68) | 1.15 (0.85–1.57) | 1.08 (0.79–1.47) | 1.07 (0.78–1.45) | 1.03 (0.75–1.41) |
| C-3600 | 1.12 (0.85–1.50) | 1.10 (0.82–1.46) | 1.06 (0.79–1.42) | 1.06 (0.79–1.42) | 1.04 (0.77–1.39) | 1.06 (0.79–1.44) | 1.07 (0.80–1.46) |
| C-1900 | 0.72 (0.49–1.04) | 0.88 (0.60–1.28) | 0.98 (0.66–1.41) | 0.99 (0.69–1.42) | 1.08 (0.73–1.52) | 1.07 (0.73–1.52) | 1.10 (0.76–1.57) |
| N-7820 | 0.43 (0.28–0.64)^**^ | 0.53 (0.35–0.80)^**^ | 0.62 (0.41–0.93)^*^ | 0.61 (0.41–0.91)^*^ | 0.61 (0.41–0.91)^*^ | 0.57 (0.38–0.85)^**^ | 0.58 (0.39–0.86)^**^ |
| B-1000 | 0.51 (0.35–0.74)^**^ | 0.51 (0.35–0.74)^**^ | 0.62 (0.42–0.90)^*^ | 0.61 (0.41–0.89)^*^ | 0.68 (0.47–0.98)^*^ | 0.69 (0.47–1.00) | 0.71 (0.48–1.01) |
| **Anxiety only** |  |  |  |  |  |  |  |
| P-8300 | 2.23 (1.11–4.69)^*^ | 2.82 (1.28–6.88)^*^ | 2.25 (0.98–5.40) | 2.23 (0.97–5.23) | 2.49 (1.09–6.21)^*^ | 2.28 (0.99–5.60) | 2.24 (0.97–5.37) |
| Q-8400 | 1.30 (0.58–2.80) | 1.45 (0.63–3.51) | 1.27 (0.55–3.10) | 1.27 (0.54–3.12) | 1.21 (0.52–2.90) | 1.23 (0.52–3.03) | 1.22 (0.51–2.99) |
| G-6000 | 2.70 (1.37–5.53)^**^ | 2.52 (1.24–5.36)^*^ | 2.46 (1.20–5.31)^*^ | 2.23 (1.10–4.80)^*^ | 2.03 (1.00–4.36)^*^ | 1.77 (0.88–3.86) | 1.77 (0.88–3.85) |
| E-0000 | 1.38 (0.63–2.98) | 1.33 (0.62–2.91) | 1.46 (0.66–3.27) | 1.44 (0.66–3.11) | 1.44 (0.66–3.20) | 1.35 (0.63–3.00) | 1.31 (0.61–3.04) |
| G-5400 | 1.59 (0.75–3.42) | 1.52 (0.72–3.47) | 1.48 (0.68–3.42) | 1.40 (0.63–3.25) | 1.37 (0.63–3.06) | 1.25 (0.58–2.87) | 1.24 (0.57–2.89) |
| C-3900 | 1.66 (0.79–3.55) | 1.59 (0.77–3.44) | 1.72 (0.81–3.78) | 1.57 (0.74–3.33) | 1.45 (0.71–3.09) | 1.44 (0.68–3.18) | 1.46 (0.71–3.25) |
| C-3600 | 0.73 (0.29–1.73) | 0.68 (0.28–1.60) | 0.68 (0.27–1.67) | 0.68 (0.27–1.58) | 0.67 (0.26–1.64) | 0.74 (0.29–1.81) | 0.73 (0.29–1.73) |
| C-1900 | 1.21 (0.55–2.63) | 1.26 (0.57–2.89) | 1.50 (0.67–3.50) | 1.52 (0.68–3.34) | 1.67 (0.75–3.75) | 1.72 (0.76–3.91) | 1.64 (0.77–3.85) |
| N-7820 | 1.11 (0.50–2.45) | 1.24 (0.55–2.94) | 1.48 (0.65–3.47) | 1.46 (0.64–3.38) | 1.46 (0.65–3.41) | 1.27 (0.55–2.94) | 1.23 (0.53–2.95) |
| B-1000 | 0.74 (0.31–1.70) | 0.69 (0.29–1.66) | 0.91 (0.37–2.28) | 0.90 (0.36–2.14) | 1.02 (0.42–2.47) | 1.07 (0.42–2.66) | 1.03 (0.41–2.53) |

Note: The covariates were sequentially added to the model. Results were shown by odd ratio with 95%CI. A Bayesian multinomial mixed-effects logistic regression was fitted for the cormobid depressive-anxiety symptoms.

C-1900: leather, fur, feather and their products, and footwear manufacturing industry; C-3600: automotive manufacturing industry; C-3800: electrical equipment manufacturing industry; E-0000: construction industry; B-1000 (reference): non-metallic mineral mining industry; C-3900: computer and communication equipment manufacturing industry; Q-8400: health care and social work industry; P-8300: education industry; N-7820: environmental sanitation industry; G-5400: transportation industry; G-6000: express delivery and food delivery industry.

^*^: <-.05; ^**^: <0.01
