# Supplementary material for: Depressive and Anxiety Symptoms and Their Comorbidity Across Industries: Multi-Industry Cross-Sectional Study in China
Source: JMIR Public Health Surveill. 2026 Aug 21;12:e91782. doi: 10.2196/91782 (PMC13495997; doi:10.2196/91782)
Supplement: Supplementary file 3 [file publichealth-v12-e91782-s003.docx]

STROBE Statement—Checklist of items that should be included in reports of ***cross-sectional studies***

|  | Item No | Recommendation | Location (or reason for not reporting) |
| --- | --- | --- | --- |
| **Title and abstract** | 1 | (*a*) Indicate the study’s design with a commonly used term in the title or the abstract | Title and first sentence of Methods of Abstract |
|  |  | (*b*) Provide in the abstract an informative and balanced summary of what was done and what was found | Abstract: Methods, Results |
| Introduction | | | |
| Background/rationale | 2 | Explain the scientific background and rationale for the investigation being reported | Introduction: line 82-110 |
| Objectives | 3 | State specific objectives, including any prespecified hypotheses | Introduction: line 111-118 |
| Methods | | | |
| Study design | 4 | Present key elements of study design early in the paper | Methods: Study design, line 121-123 |
| Setting | 5 | Describe the setting, locations, and relevant dates, including periods of recruitment, exposure, follow-up, and data collection | Methods: Survey Development, line 126-137 |
| Participants | 6 | (*a*) Give the eligibility criteria, and the sources and methods of selection of participants | Methods: Participant Recruitment, line 139-156 |
| Variables | 7 | Clearly define all outcomes, exposures, predictors, potential confounders, and effect modifiers. Give diagnostic criteria, if applicable | Methods: Survey Measures, line 158-176  Discussion section: Limitations, line 406-409 |
| Data sources/ measurement | 8* | For each variable of interest, give sources of data and details of methods of assessment (measurement). Describe comparability of assessment methods if there is more than one group | Methods Survey Measures |
| Bias | 9 | Describe any efforts to address potential sources of bias | Methods:Statistical Analysis, line 181-185  Discussion section: Limitations. |
| Study size | 10 | Explain how the study size was arrived at | Methods: Participant Recruitment |
| Quantitative variables | 11 | Explain how quantitative variables were handled in the analyses. If applicable, describe which groupings were chosen and why | Methods: Survey Measures and Statistical Analysis |
| Statistical methods | 12 | (*a*) Describe all statistical methods, including those used to control for confounding | Methods: Statistical Analysis |
|  |  | (*b*) Describe any methods used to examine subgroups and interactions | Methods: Statistical Analysis |
|  |  | (*c*) Explain how missing data were addressed | Methods: Statistical Analysis |
|  |  | (*d*) If applicable, describe analytical methods taking account of sampling strategy | Methods: Statistical Analysis, line 184-185 |
|  |  | (*e*) Describe any sensitivity analyses | Methods: Statistical Analysis, line 183-184 |
| Results | | | |
| Participants | 13* | (a) Report numbers of individuals at each stage of study—eg numbers potentially eligible, examined for eligibility, confirmed eligible, included in the study, completing follow-up, and analysed | Results: Participant Characteristics; Table 1 |
|  |  | (b) Give reasons for non-participation at each stage | Not applicable |
|  |  | (c) Consider use of a flow diagram | Not applicable |
| Descriptive data | 14* | (a) Give characteristics of study participants (eg demographic, clinical, social) and information on exposures and potential confounders | Results: Participant Characteristics; Table 1 |
|  |  | (b) Indicate number of participants with missing data for each variable of interest | Not applicable |
| Outcome data | 15* | Report numbers of outcome events or summary measures | Results: line 224-234, Table 2 |
| Main results | 16 | (*a*) Give unadjusted estimates and, if applicable, confounder-adjusted estimates and their precision (eg, 95% confidence interval). Make clear which confounders were adjusted for and why they were included | Results: Tables S1, S2, S3, S4, Figure 2 |
|  |  | (*b*) Report category boundaries when continuous variables were categorized | Methods: line 161-168 |
|  |  | (*c*) If relevant, consider translating estimates of relative risk into absolute risk for a meaningful time period | Not relevant |
| Other analyses | 17 | Report other analyses done—eg analyses of subgroups and interactions, and sensitivity analyses | Results: Table 3 |
| Discussion | | | |
| Key results | 18 | Summarise key results with reference to study objectives | Discussion: Principal Findings, line 290-375 |
| Limitations | 19 | Discuss limitations of the study, taking into account sources of potential bias or imprecision. Discuss both direction and magnitude of any potential bias | Discussion: Limitations, line 398-409 |
| Interpretation | 20 | Give a cautious overall interpretation of results considering objectives, limitations, multiplicity of analyses, results from similar studies, and other relevant evidence | Discussion: Conclusions |
| Generalisability | 21 | Discuss the generalisability (external validity) of the study results | Discussion: Limitations, line 376-393 |
| Other information | | | |
| Funding | 22 | Give the source of funding and the role of the funders for the present study and, if applicable, for the original study on which the present article is based | Discussion: Funding, line 399-402 |

*Give information separately for exposed and unexposed groups.

**Note:** An Explanation and Elaboration article discusses each checklist item and gives methodological background and published examples of transparent reporting. The STROBE checklist is best used in conjunction with this article (freely available on the Web sites of PLoS Medicine at http://www.plosmedicine.org/, Annals of Internal Medicine at http://www.annals.org/, and Epidemiology at http://www.epidem.com/). Information on the STROBE Initiative is available at www.strobe-statement.org.
